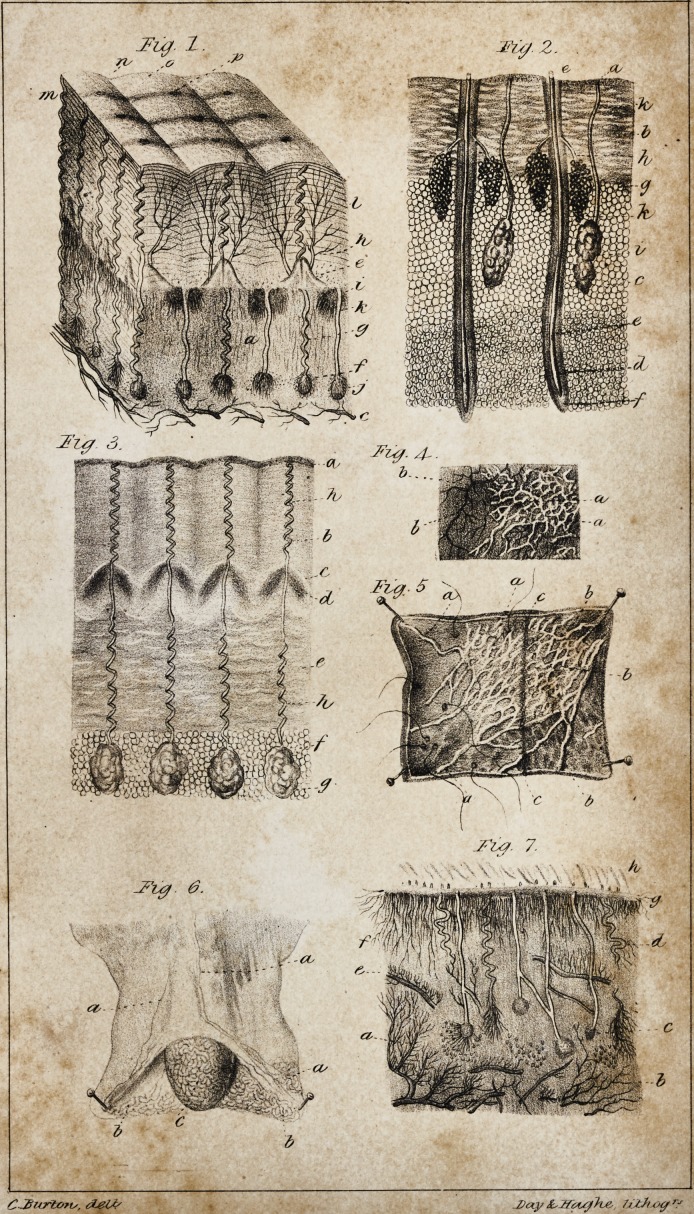# New Enquiries on the Structure of the Skin

**Published:** 1836-10

**Authors:** 


### Art. VI.

1. *Nouvelles Recherches sur la Structure de la Peau.* Par M. G. Breschet et M. Roussel de Vauzème.—*Paris*, 1835. Pp. 121.
*New Enquiries on the Structure of the Skin.* By M. G. Breschet and M. Roussel de Vauzème.

2. *Vergleichende Untersuchungen über die Haut des Menschen und des Haus-Saügethiere, besonders in Beziehung auf die Absonderungsorgane des Haut-Talges und des Schweisses.* Von Gurlt.—*Berlin*, (*Müller's Archiv.*) 1835.
*On the Comparative Anatomy of the Skin of Man and the Domestic Mammalia, more particularly as it regards the Organs of the Oleaginous Secretion and of the Perspiration.* By Professor Gurlt.

3. *Ueber die menschliche Epidermis.* Von Dr. Alphons Wendt.—*Berlin*, (Müller's Archiv.) 1834.
*On the Human Epidermis.* By Dr. Alphons Wendt.

Few organs have afforded a wider field for the range of anatomical fancy than the external integument. The differences of opinion which have existed respecting its structure may be ascribed (in addition to the difficulties attending on all microscopic investigations,) to the want of acquaintance of anatomists with facts previously recorded; to conclusions drawn from false physiological analogies; to hasty inferences from imperfect observations; and perhaps, above all, to the manner in which the skin has been hitherto examined.

Had the works of Malpighi, to which we are indebted for the first minute analysis of the texture of the skin, together with those of Ruysch, Albinus, Kaau, Sacretaire, Winslow, and others, met with the attention which they deserve, many supposed discoveries would have been found to be recorded facts, and many of the errors and misrepresentations which characterize the works of later anatomists would have been avoided. The results of the enquiries of the French and German authors before us tend in some particulars to reconcile the diversities of opinion which have hitherto existed, although in others it appears probable that they have but added to the list, without approaching nearer to the truth.

The French authors have entered largely into the history of their subject, but without giving a due share of their attention to facts recorded by the older anatomists; and they have consequently claimed the merit of discovery to an extent beyond that which they deserve. A brief allusion to the opinions which have been entertained on the subject of the structure, origin, and functions of the constituent parts of the skin, may not inappropriately precede the consideration of the works before us.

The epidermis (meaning by the term to include the whole of that structure which is situated external to the dermis, and its papillary eminences,) has by some anatomists been described as scaly, by others as composed of layers placed over one another; opinions have varied as to the number of these layers. It has been maintained by some that the cuticle and rete mucosum are distinct membranes, whilst others have asserted that the difference consists only in the greater density of the former. At one time the epidermis has been considered as vital and organized; at another, as destitute of any of the characteristics of living matter. Its porosity has been regarded as certain by one class of anatomists, (who do not, however, agree as to the character and disposition of the pores;) by another, this has been denied, and particularly in later times, since the property of imbibition has been ascertained to belong not only to living, but also to dead membranes. It has even been maintained that, so destitute is it of pores, that its outer layer is continued over the entire surface of the hair. The appearance which the late Mr. Chevalier described by the term "velamina;" and that of certain bodies existing within the epidermis, to which he gave the name of "inter-epidermal glands," were probably the consequence of mistaken notions respecting the functions of the skin, as well as of the mode in which his investigations were conducted.

The origin of the epidermis has given rise to a variety of opinions. Thus, it has been considered as an efflorescence of the papillæ or dermis; as an effect of the compression of the air; as a union of the exhalent vessels; as an agglomeration of the globules of the blood, deprived of their fibrin and dried; as a result of the oxidation of the rete mucosum; and some of those who have maintained its distinction into cuticle and rete mucosum regarded these, together with the dermis, as an expansion and greater development of the meninges of the brain, conveyed thither as coverings of the nerves.

Its colour has likewise been variously stated, as well as the causes to which this was to be attributed. Among those who have considered the rete mucosum as a distinct membrane, its nature has been variously described. Thus it has been termed a fine cellular membrane; a plexus of veins, arteries, and lymphatics; a vascular mucous network; a compound of several layers, and a tissue of the same nature as the grey substance of the brain. The existence of absorbents in the epidermis has always been hypothetical; the filaments seen on separating it from the dermis having by some been imagined to perform this office, whilst others have ascribed to them nervous or exhalent functions, or simply that of connecting the epidermis with the parts on which it rests. The greatest uncertainty has always prevailed as to the organs appropriated to both the insensible perspiration and sweat, and it has been a question whether these were to be considered as distinct functions or as consisting only in difference of degree. The functions of absorption, secretion, and of perspiration; the property of erectile tissues, have all been ascribed to the papillæ; but the opinion of their discoverer, Malpighi, as well as that more generally entertained, has been that they are chiefly the organs of sensation. More recently they have been considered, in other animals, as aborted hairs.

The dermis has been described as an inextricable tissue. Its vascularity, porosity, and the course of its nerves towards the papillæ have been long known; but, beyond the very evident characteristics of its structure, little until the present time has been ascertained. The structure of the sebaceous glands and hair follicles, has generally been regarded as that of a simple sack, and the existence of the former independently of the latter has been denied.

The researches of the present authors may be considered as having decided some of these doubtful points; and it is satisfactory that, with regard to the existence and characteristics of certain organs, their investigations have led to corresponding results, although their opinions differ in several important particulars.

The attention of MM. Breschet and Roussel de Vauzème has been directed to the structure of the dermis; to the organs of sensation, perspiration, and absorption; to the sources whence the epidermis is derived, and to its structure and the cause of its various colours. M. Gurlt has described with greater minuteness the structure of the perspiratory apparatus; has examined the skin, with a view to confirm or disprove the opinions of the French authors with respect to the existence of organs appropriated exclusively to the formation of the epidermis and its colouring matter; and has described the sebaceous glands and follicles of the hair. The paper of M. Wendt contains some additional observations on the sudorific organs, and on the origin and structure of the hair.

In our examination of the works before us, we shall follow the arrangement adopted by MM. Breschet and Roussel de Vauzème.

The following is their division of the various parts constituting the skin.*

I. Dermis (Pl. I. Fig. 4, *a*; Pl. II. Fig. 1, *a*.)

II. Papillæ (Pl. I. Fig. 3, *a*; Pl. II. Fig. 1, *e*.)

III. Sudorific organs (Pl. I. Fig. 4, *b*, *c*; Pl. II. Fig. 1, *f*, *g*.)

IV. Inhalent organs (Pl. I. Fig. 8, 9, 10; Pl. II. Fig. 1, *h*.)

V. Mucific apparatus (Pl. I. Fig. 11, *a*, *b*, *c*; Fig. 13, *e*, *g*; Pl. II. Fig. 1, *j*.)

VI. Cororific apparatus (Pl. I. Fig. 13, *a*; Pl. II. Fig. 1, *i*.)

The secretion of the last two constitutes the epidermis; consisting of the pretended rete mucosum of Malpighi and the cuticle.

We shall employ the term epidermis, (instead of horny tissue, horny matter, epidermic layers, which are used synonymously by the French authors,) to signify every part which is situated above the epidermis and papillæ, exclusive of the various organs which it contains. The word cuticle is only applied to the external layer of the epidermis. The skin of the heel is recommended as the most favorable for examination.

CHAPTER I. *The Dermis.* For the inspection of its minute structure, a portion should be selected which is either injected by

* All the references are to the Plates in the present Number, which are accurate copies from the originals of Breschet and Gurlt.—EDS.

blood or artificially; for, when the dermis is white, it is impossible to distinguish the vascular and other tissues. As thin a slice as possible should then be cut off with a very sharp knife, parallel to or at right angles with its furrows. This is then to be placed on a piece of glass, in the form of a lens, and illuminated with reflected light. This process is sufficient for the examination of all the structures of the skin; but it requires some precautions. The dermis must be somewhat dried in the air; for, if soft, it cannot be cut in such a manner as to allow its parts to be distinguished. From the dermis thus prepared, the secreting organs and nerves may be isolated by means of sharp curved needles. It is a dense fibrous envelope of the capillary bloodvessels, lymphatics, nervous filaments, and parenchyma of other organs. It is white, but readily coloured by cadaverous transudations and injections; of a rosy tint in some individuals, from the fullness of its bloodvessels. It never shares in the colouring matter of the epidermis. Beneath it are easily seen the vessels and nerves which penetrate it, and openings for glandular bodies. The absorbent vessels escape from it. Externally (Pl. I. Fig. 1, *j*,) it is elevated into symmetrically arranged, conical eminences, disposed in straight or somewhat curved lines, (Pl. I. Fig. 1, *k*,) separated by furrows, (Pl. I. Fig. 1, *m*,) running in the same direction. Each prominent line is divided transversely by small fissures, at the bottom of which is seen a hole, (Pl. I. Fig. 1, *t*.) On its external surface, the dermis terminates in a membrane, which appears to confound itself with the papillary organs and with those for the secretion of the colouring matter. This external surface is perforated for the passage of the secreting and absorbing apparatus. The history of the dermis must be completed when speaking of the organs with which it is connected. It may be isolated by dissection as far as its external surface, where it is so confounded with the parenchyma of the secreting and sensitive apparatus that it is impossible to distinguish them. In addition to the organs here mentioned, as existing in the internal surface of the dermis, Gurlt mentions the hair follicles which project into the subdermic tissue.

Chapter ii. *The Papillæ* (*appareil nevrothèle, or mammillary nervous apparatus*. The oblique course of the nerves in the substance of the dermis renders it necessary that their points of entrance and exit should be ascertained previous to making an attempt to follow them; otherwise they are divided by any incision. But, with some care, very thin bundles of pulpy filaments, which penetrate the bases of the papillæ, may be distinguished and isolated, between the excretory canals on the surface of the dermis, (Pl. I. Fig. 5.) These papillæ (Pl. I. Fig. 3, *a*, Fig. 12, *b*, Pl. II. Fig. 1, *e*,) arranged in a continuous series, are ordinarily bifid or trifid; separated transversely by the intervals which give passage to the sudoriferous canals, and longitudinally by the furrows from which the epidermic matter escapes. Their form is that of a small cone, the base of which vanishes in the cutis; the summit terminating in a blunt point. Each papilla penetrates the epidermis, so that the internal surface of the latter represents exactly the number and arrangement of the former, (Pl. I., Fig. 1, *f.*) When forcibly separating these two portions of the skin, the papillæ adhere to the dermis by their bases, whilst the epidermis is readily detached from them. Their direction is oblique. Besides a neurilema which they derive from the dermis, (Pl. I., Fig. 5,) the epidermis furnishes them with a proper sheath, (Pl. I., Fig. 6.) Their summit is not pierced by any aperture. In the skin of the whale, they are remarkably developed, and differ but in form from those of the human subject. The length of the papillæ varies with the thickness of the skin in different parts of the same animal. In the whale, their bases, confounded with the dermis by a bundle of radicles, is marked by striæ, which insensibly disappear towards the termination of these bodies; the extremity being somewhat enlarged. They end not far from the surface of the epidermis. Their external surface is white, pearly, opaque. Magnified to an extreme degree, the body of the nerve presents, through the neurilema, slight undulated striæ, which, coming from their bases, become less marked in proportion as they creep towards the swollen extremity, where they appear to terminate in concentric semicircles. This surface is smooth and even. No process is detached to communicate with the neighbouring parts. After every examination, nothing but a white dense tissue, quite analogous to the nervous, has been seen. For a long time the striæ, apparent externally, were not separable into bundles. With some trouble an internal nutritive vessel was found. On making a transverse section of an injected papilla in the human skin, at least two vessels are found, which appear to unite so as to form a loop. The centre of the papilla seems to contain a pulpy matter. The papillæ of the whale, compared with the human papillæ, hitherto ill observed, leave no longer room for doubt as to their structure and sensitive functions. The papillæ of the negro do not differ from those of the white. MM. Breschet and Roussel de Vauzème dissent from the common opinion that the proper papillæ of the tongue are the organs of the sense of taste; on the ground, that in some animals (for instance, the ox,) they are covered by a horny case, more or less thick, which opposes the functions attributed to them; but that in their interstices, beneath a thin epithelium, a similar papillary structure to that of the skin is observable. Here they would exclusively place the sense of taste. The other papillæ they conceive to be but accessory, and rather constituting an organ of touch; their number being too limited for a nervous system, such as that of taste, and their structure assimilating them more or less to horns, hoofs, &c. Their direction, from before backwards, appears appropriated to draw the food into the pharynx, to multiply its points of contact with the tongue, and render it more perceptible to the sensitive papillæ of the interstices. They may also serve for a special sense combined with that of taste; and possibly the two orders of papillæ may differ only in the disposition of their envelope. There is certainly something rather confused and indefinite in these remarks on the papillæ of the tongue; but that its proper papillæ are not organs of taste we should hesitate to decide; because, until we know in what the sense of taste differs from that of touch, we cannot say that the structure in the ox is not adapted to it; and, in the absence of direct evidence to the contrary, we should infer that what constituted a peculiarity in the nervous structure of the tongue was subservient to its proper function. We know also that the tongue is a very delicate tactile organ, and the French authors mention that a similar papillary structure to that of the skin, and which is also found in the interstices of the proper papillæ of the tongue, to which they ascribe the sense of taste, is found in the mucous membrane of the nose and throat.

They proceed to remark, that the papillæ of the dermis are always found in an area circumscribed by the secreting organs of the epidermis. There can be no doubt of the purely nervous character of the papillæ, if that part of the dermis be examined where they end; the same order existing in the interior of the dermis as at its surface. The prominent lines correspond to the nerves; their interstices to the sudoriferous canals and inhalent vessels; in the bottom of the furrows is found the source of the epidermis. If the tactile sense is refused to the papillæ, what other organ in the skin can be said to possess this function? The nerves which supply the skin may be seen in three different conditions: (1,) in the subcutaneous tissue, where they do not differ from other nerves coming from the spinal marrow; (2,) within the substance of the dermis, where they become soft, flexuous, capillary; (3,) on its external surface, where they are transformed into symmetrical papillary eminences. It is probable that the nerves detach their neurilema on arriving at the dermis. When, however, they reach the external surface of the dermis, they are certainly covered by its external membrane. Thus the sense of touch is provided, as those of sight and hearing, with a peculiar apparatus, consisting of—A. Principal part: (1,) tactile nerve terminating in a blunt point. B. Accessory or protecting parts: (2,) dermis, containing the nerve within its substance; (3,) papillary neurilema, furnished by the dermis; (4,) proper sheath; modified, and of epidermis; the organ of protection; (5,) thin layer of epidermis covering the sheath of the papilla, and indispensable to the exercise of touch. If any of these is wanting, or undergoes certain modifications, touch cannot be exercised. It is evident that the dermis, the neurilema, the proper sheath of the epidermis, are to the tactile nerves that which the complicated apparatus of the organs of sight and sound are to the optic and auditory nerves.

The anastomotic termination of the nervous filaments, already described as having been observed in the papillæ of the whale, is brought forward as confirmatory of the opinion already advanced by MM. Prevost and Dumas, on the mode in which the ultimate termination of nerves is effected. The appearance of concentric loops at the points of the papillæ, although the possibility of its being an optical illusion is admitted, has been so frequently seen, that MM. Breschet and Roussel de Vauzème believe that they have discovered the truth. Against the opinion advanced by Steller, and to which that of Blainville somewhat approximates, that the papillary bodies in the cetaceæ are agglutinated hairs, the following characteristics of the papillæ are considered as conclusive: Their whiteness; their olivary termination within the epidermis; the communication of their bases with the nerves of the cutis, where the papillæ have neither a fixed termination nor a bulbous enlargement, and the filamentous appearance which is observed beneath the microscope. Rapp considers the papillæ as true vessels, that they very much resemble the flocculi of the duodenal mucous membrane of the mammiferæ and of many birds, and that they are the secreting organs of the matter constituting the rete of Malpighi. This function of secretion is denied to them by the French authors, who regard them as exclusively nervous organs.

It would be far to exceed our limits were we to enter with minuteness into the various opinions which have been entertained respecting the nature of the cutaneous papillæ. There appears, however, much reason to believe that the French authors have somewhat limited their functions in considering them exclusively nervous, and that, by ascribing to them the simple function of secretion, M. Rapp has fallen into an opposite error. We are disposed to believe that they are both organs of sensation and of secretion. The chief grounds of objection to the opinions of MM. Breschet and Roussel de Vauzème are, that the papillæ are far more vascular than they are disposed to admit, and that these vessels are probably partly the source of the epidermis; a function which they have ascribed to other organs, the existence of which is doubtful.

Largely as they have entered into the history of their subject, they have entirely neglected to notice the opinions of some of the older anatomists, whose discoveries have in many points preceded their own. Malpighi was the first to describe the papillary bodies, in his work "De externo Tactus Organo," 1686. He considered them as organs of touch, but does not appear to have had any very accurate knowledge of them. Ruysch, in describing a drawing in the first number of the Thesaurus Anatomicus, says, "Tab. 4, Fig. 9, represents a portion of the nipple of the whale's udder, the cutaneous papillæ of which I have separated into their component parts in pure water; that is, each papilla consists of many nervous fibres. . . . But, on account of the intimate adhesion of their fibres, I have never been able to effect this dissolution of the cutaneous papillæ in man."*

* Tab. i. fig. ix. Theas. Anat. i. repræsentat portionem papillæ uberis Balænæ cujus papillæ cutaneæ a me dissolutæ sunt in aqua simplici, id est, singulæ papillæ ex multis fibris nervosis constitutæ quidem sunt. . . . In homine autem hanc dissolutionem papillarum cutanearum nunquam peragere potui utpote fibris sibi invicem firmius cohærentibus.—*Theasaurus Anatomicus Primus.*

Kaau dissected the subcutaneous nerves as far as the papillæ. In objecting to the notion that the three constituent parts of the skin were formed by the neurilemas of the nerves, which had followed their course from the meninges of the brain, he says, "These (the subcutaneous nerves), in proportion as they approach the cutis, become increasingly delicate, until there arise from them very small and innumerable branches, which enter the cutis, and approach the papillæ in which they are arranged. That they are, as far as this, still enclosed in their investing tunics, I am convinced; for I have followed them to this point, by means of a microscope and a very fine needle, the contact of which they bear in this situation."* MM. Breschet and Vauzème are unable to decide whether the subcutaneous nerves detach their neurilema on entering the dermis, and suppose some such change to take place as that which happens to the optic nerve when entering the sclerotic coat. But, as Kaau observes, unless the filaments within the dermis were still enclosed in their coverings, it would scarcely be possible to follow their course. Sacretaire has distinctly mentioned the union of the extremities of the nervous filaments constituting the papillæ. Speaking of those in the palms of the hands, he remarks: "In these parts are situated certain corpuscules, composed of four, five, or more filaments, as it were, arising from the cutis, connected together by their extremities, and arranged in a definite order."†

The French authors speak of the blood-vessels of the papillæ as exclusively appropriated to their own nutrition; all the products which are found external to the dermis and papillæ being secreted in the interstices of these bodies. We shall hereafter notice their opinions on the source of the epidermis; but, as the extremely vascular nature of the whole surface on which the epidermis rests is a main reason for the difference of our opinion, we must state our doubts respecting what they have said of the papillæ. They say that, in these bodies in man, there are at least two blood-vessels which appear to unite and form an arch; that their external surface is white, pearly, opaque; and that, notwithstanding all their endeavours, they have been only able to detect a tissue entirely analogous to that of nerves. They mention likewise that an external membrane, supplied by the outer surface of the dermis, is continued over the papillæ. This description is quite at variance with the general opinion, as well as with the appearance of well-injected preparations. Can it be supposed that the French authors have been unsuccessful in their injections? In some injected prepara-

* Hi (nervi subcutanei) quoque propiores cuti, eo fiunt teneriores, donec ex hisce, minimi, innumeri adsurgunt ramuli, qui cutim intrant, ad papillas tendunt, et in iis ordinantur. Imo vero has huc usque servare, quibus investiuntur tunicas affirmo. Eo usque prosecutus sum, microscopio et acu tenuissima, cujus attactum et in hoc loco ferunt. (*Kaau, Perspiratio dicta Hippocratici.* 1738.)

† In his enim partibus locantur corpuscula quædam, composita ex quatuor, quinque vel plurimis quasi filamentis e cute surgentibus, suis extremis inter se connexis, certoque ordine disponuntur. (*Dissertatio Med. Inaug. de Com. Corp. Hum. Integ.* 1727.)

tions, it will be seen that some of the papillæ on the ends of the fingers terminate in white points, that others are entirely coloured by the fluid; and into other parts of the skin the injection has passed in such a manner as to render the papillæ, as well as their interstices, uniformly coloured. It would be tedious to quote authorities in support of their high vascularity. Malpighi, Ruysch, Albinus, Kaau, Sacretaire, Haller, and, in later times, Bichat, Beclard, Gaultier, Chevalier, Rapp, and others, have all asserted this fact.

With regard to the external covering of the papillæ, which is said to be derived from the external surface of the dermis, the French authors speak of it as white and fibrous; but they say nothing concerning its vascularity.

The anastomotic appearance of the extremities of the nervous filaments, it is admitted, may arise from an optical illusion; and although this mode of termination is said to be in concentric semicircles, the drawings do not represent a concentric arrangement.

MM. Breschet and Roussel de Vauzème have not particularly noticed the distinction between the common sensibility and the tactile properties of the skin. They say that tact is exercised by the thousands of organs communicating together by means of a nervous plexus, extended over the whole surface of the body, from one papilla to another; and that it is this lateral correspondence of all the papillæ, or, in other words, the organ of touch established in the skin, which render it the most sensible part of the whole body. The existence of the peculiar apparatus described is said to be essential to the exercise of the function, which is destroyed by the absence or alteration of any one of its constituent parts. But, on the following grounds, there is reason to doubt whether this entire apparatus is necessary for either common sensation alone, or for it in combination with tact. M. Gurlt observes, that the papillary structure appears to be entirely wanting on the hairy scalp. The French authors have described it as existing in the heel. From the experiments of Professor Weber, confirmed by Dr. Allen Thompson, (Edinb. Med. and Surg. Journal, No. 116,) it appears that the heel possesses a tactile sense very little superior to that of the scalp; and that, in the latter, this sense is more acute than in various parts of the body; although MM. Breschet and Roussel de Vauzème have spoken of the papillæ and their coverings as existing over the whole body. There appears, therefore, reason to suspect that, even if the compound structure which has been described actually exists, it is confined to certain localities, and that both the function of tact and that of common sensation may be carried on by means of some much more simple apparatus.

Chapter iii. is devoted to the *Sudorific Organs and their Ducts (appareil diapnogène et canaux sudorifères ou hidrophores).*

The exhalent organ consists of a secreting parenchyma and an excretory duct. The parenchyma is situated in the substance of the dermis, surrounded by numerous capillaries which are attached to it. Its form is that of a somewhat distended sac, whence a spiral duct issues, which, traversing the dermis, escapes from it at the transverse fissure situated between the papillæ; thence it passes obliquely and in a spiral form through the epidermis, on the surface of which its termination is indicated by a slight depression or pore in the prominent lines of the cuticle. In consequence of its spiral course, the duct opens on the surface obliquely, and the aperture is closed by the contact of the upper and lower parietes of the tube. In examining the transudation of the sweat, the first drop is seen to be preceded by an elevation of the cuticle, in the manner of a valve. At the exit of these canals from the dermis, they are accompanied by an inhalent vessel, which enters into the infundibulum of the papillæ. The spiral form of these tubes accounts for the fact that the cuticle has always appeared imperforate. Thus, when the epidermis is torn from the dermis, the lacerated ducts retract and stop up the opening. In the skin of the heel, by a successive removal of all the layers of the epidermis, these tubes may be followed from the surface to the intervals of the papillæ; the situation of the tubes being indicated by an oblique depression produced by the contact of its sides; and, by gentle pressure, a drop of sweat may be made to exude from this orifice. The most convincing proof that these spiral bodies are ducts is the escape of sweat from the pores of the palms of the hands and points of the fingers. (Pl. I. Fig. 4; Pl. II. Fig. 1, *f*, *g*.)

M. Gurlt has given a more minute account of the structure of the sudorific glands, having examined them in various domestic mammalia. He has described also certain peculiarities in these organs existing in the same individual, which tend to correct some errors into which it appears probable that the French authors have fallen. He says, that they lie deeper in the substance of the dermis than the sebaceous glands, and extend more plentifully beyond into the adipose tissue. They exist in every part of the skin, but differ, in the different situations, in size, form, and also partly in texture. In the human palm (Pl. II. Fig. 3, *g*,) and sole, they are larger than elsewhere, and of a roundish oval shape; in the skin of the head, they are more oblong, (Pl. II. Fig. 2, *i*.) They are readily recognized, by the naked eye, in the loose cellular tissue beneath the cutis of the parts of generation of the horse. In the ox, the glands are very small and round, and everywhere uniform in shape and size. Other varieties are noticed as existing in different animals. In regard to texture and colour they are of two kinds. In man, the horse, sheep, swine, and dog, (on the soles,) they consist of a canal or tube many times doubled on itself; and in this respect they greatly resemble the texture of the testicle: in the ox they are round, and in the hairy parts of the dog, long, small sheaths, without any trace of windings or foldings. In most cases they are colourless and almost transparent; on the genitals of the horse they are brown-coloured, which is owing to the presence of small, brown granules, contained in the twisted tube. In those of the dog's foot there are likewise granules to be seen, but they are almost colourless; at least, the gland does not put on any coloured appearance from their presence. In the representations given of these organs by the French authors, the glands are too small in proportion to their canals. At the superficial termination of these ducts, it is probable that the epidermis dips into them; as, in animals with coloured epidermis, we observe the same colour at the entrance of the perspiratory pores, and only at some distance do they become colourless and transparent; moreover, they have entirely the same texture as the epidermis. In the human hand and foot, the ducts, in passing outwards through the dermis amid the tactile papillæ, are very slightly or not at all spiral; but, in the epidermis, they form a greater or less number of spiral windings, which are remarkably well seen in a piece of skin which has been indurated and rendered transparent by means of Liq. pot. carb. (Pl. II. Fig. 3, *h*, *h*.) The number of these windings increases with the diameter of the cuticle. For the representation of these glands, recent skin was always employed. In no other part of the human skin do the perspiratory canals exhibit such fine spiral windings; and, among domestic animals, they are spiral only in the sheep; in all others, they are merely serpentine.

Wendt has also remarked, that these vessels, in the number of their windings and in their direction through the skin, vary in different parts of the body; that in the thinnest parts of the skin they may make but half a wind; and that, in such situations, they are less regularly placed than in the hand and foot. He found that, in the right hand, these spiral tubes were bent from left to right, and in the contrary direction in the left hand.

The merit of the discovery of this sudorific apparatus, although it has given rise to some dispute, appears to be attributable to MM. Breschet and Roussel de Vauzème. But there can be no doubt that some of the older anatomists were aware of the existence of the glandular part of this apparatus. Malpighi speaks of glands situated beneath the dermis, and opening by excretory ducts. These apertures he terms "ora sudoris." That by these, as has been asserted, he could not have meant the sebaceous glands, is evident from his having described them as existing at the points of the fingers, (*de externo tactus organo.*) From the description of these organs which is given by Winslow, and which corresponds so exactly with that of Gurlt, there can be no doubt that he was aware of their existence and character. Speaking of these glands, he says, "Feu M. Duvernay a montré à l'Academie Royale des Sciences, assez clairement, la structure de quelques uns de ces glandes cutanés, qui paroissent comme des *circonvolutions des petits intestins*, chargés de vaisseaux capillaires. (Compare this description with Gurlt's drawing. Pl. II. Fig. 3, *g*, and Fig. 2, *i.*) The mode in which the epidermis was separated from the dermis will account for the spiral ducts not having been detected in the latter part of their course, and this form is often absent in the dermis. Thus, the French authors can scarcely be considered as the discoverers of the glands, although the character of their ducts does not appear to have been previously described.

Chapter iv. *The inhalent Apparatus of the Integuments.* To examine this effectually, it is necessary to take a thin slice of epidermis, the most external; to select it soft, white, and somewhat friable; to place it on a piece of glass, moistened with water, and then to tear it with instruments with curved points. The inhalent canals then appear to be situated beneath the most superficial layer of the epidermis, in the form of isolated radicles, spread throughout the epidermis, and, after frequent anastomoses, penetrating the cutis by the infundibulum of the papillæ near the sudoriferous canals. All these vascular trunks, symmetrically arranged in the interstitial fissures which they traverse, communicate in the cutis, beneath the papillæ, with canals forming a common plexus, lying at a right angle with the furrows, (Pl. I. Fig. 7, *a.*) Notwithstanding all our endeavours, (say the French authors,) we have very rarely been able to see this termination of the inhalents of the epidermis. These vessels, of extreme tenuity, ramified in forming loops in a hard, elastic, resisting substance, easily break, and scarcely any thing can be seen but scattered fragments. Under the microscope, their colour is white and silvery; (Pl. I. Fig. 8, 9, 10,) through the parietes of these tubes, a species of diaphragm is here and there often observed; proving, if not an identity of structure, at least an analogy with that of lymphatic vessels or veins; sometimes they are knotted, at others smooth and uniform, and generally but little elastic. By a feeble lens, or even the naked eye, these vessels may be seen on scraping the surface of the epidermis; they are sometimes very long and dry, and resemble very fine hairs. In order to see the entrance of these vessels into the cutis, it is necessary to separate the epidermis gently, and then by a lens it may be seen that each spiral vessel is accompanied by an inhalent, and that these parts are ultimately united near the cutis. The inhalent vessel soon separates, so that the sudoriferous duct enters into the epidermic tissue by the interpapillar division, whilst the inhalent vessel diverges to the side of the epidermic partition, which corresponds to the furrows of the cutis. The microscope shows the distinctions between these two organs. The sudoriferous canal is larger and covered by little imbricated laminæ, soft, serpentine, and elastic; the inhalent vessel is smooth, silvery, straight, or slightly curved, traversed by a visible central canal, imperfectly interrupted by small partitions. If the epidermic tissue be hastily separated, the inhalent vessels are torn, and nothing remains but the spiral canals, which may be extended considerably. These are farther distinguished by the anastomoses of the inhalents, which are sometimes plexiform; a character which never belongs to the sudoriferous ducts. An experiment which would prove that these vessels have a direct communication with the arterial or venous system, and that they do not correspond to the lymphatic, is the following: If a fine injection is made into the chief artery of a limb, this injection stops at the cutis, as has always happened in our injections. If the skin be then cut (*en dédolant*), and pressure be made with the scalpel from within to without the injected part, the inhalent vessels of the epidermis become coloured, and are seen ramifying and anastomosing beneath the most superficial layer of the epidermis. The sudoriferous canals and the inhalents cannot be dissected throughout their whole extent, on account of the resisting nature of the epidermis; but the one are seen in fragments beneath a lens, the other are detected entire by means of injections. We have found these inhalents, with their distinctive characters, in the skin of the negro and of the elephant, and have recognised them in the skin of the whale, the porpoise, tortoise, and various fish. Whatever may be the colour of the epidermis, the inhalent canals, the nerves, and sudoriferous ducts are always white. We have seen these inhalent canals in all the skins which we have examined; a tissue in which, hitherto, the existence of any vessels has been denied. But the nature of these vessels may be questioned. If not absorbents, what are they? We cannot consider that the difference between their structure and that of those in the internal parts of the body is a sufficient reason for refusing to them the function of absorption. Every thing which is external to the cutis presents a peculiar appearance: the nerves by their mode of termination; the spiral sudoriferous tubes having no analogy with the other animal tissues. The apparent solidity of the absorbent vessels, their anastomoses and ramifications, appear to be appropriated to the tissue through which they have to pass. Soft and vacillating lymphatics would have been out of character with the epidermis, the nature of which is very dilatable and compressible. However, these characters belong much more to these vessels when in an empty state, than when distended by injection. They then resemble lymphatic vessels or venous capillaries. As there is no other function attributable to them, we must consider these vessels as inhalent: first, because their radicles are prolonged to the most superficial layer of the epidermis, because their texture assimilates them to lymphatics, and because, absorption being a function of the skin, we find no other organ appropriated to this function. Not having been able to see the commencement of these inhalent vessels, nor any open mouths in them, we suppose that absorption takes place by previous imbibition of the epidermic tissue. The termination of the lacteals in the intestines has not been seen. There is certainly identity both of the mode of termination and of the function of the two integuments. Great diversity of opinion has existed as to the mode in which absorption takes place through the skin. If, by pressing the injected matter from the arterial vessels, we have not effected a solution of continuity, the experiment proves that these inhalents do not communicate with the general lymphatic system, but with that of the capillaries; and this favours the opinion of Magendie, who considers the veins as the principal agents of absorption: or, if these vessels are to be considered as organs distinct from the sanguineous system, they can be considered but as varieties of the venous system, with which they are always in communication.

MM. Breschet and Roussel de Vauzème then enter at some length into the general opinions on the structure of the lymphatics. We must confine ourselves to such remarks on this part of the subject as are essential to an elucidation of their opinions. It seems to be generally admitted that apertures have not yet been found in lymphatic or lacteal vessels. The investigations of Dr. Boehn, which were noticed in our second Number, tend very much to reconcile the differences of opinion which have existed on this subject; and to them we refer for an account of the structure of the intestinal mucous membrane, which has apparently given rise to the idea that the lacteals terminated in the intestine by open mouths. The opinion of Blumenbach, that intestinal absorption took place through the medium of a laminar tissue, approaches nearly to that of the French authors on the origins of the inhalent canals.

Fohmann was the first who distinctly recognized a system of cutaneous lymphatics. From his injections, it appears that these vessels exist in so large a number that the cutis appears to be entirely formed of them. These vessels anastomose and form a network, pierce the cutis in all parts, and cover its two surfaces in such a manner that a needle could not be inserted without implicating a branch of extreme minuteness on the external surface of the cutis. This lymphatic plexus is not supplied with valves. Instead of them are seen contractions or valves of an irregular form, and so little developed as not to impede the passage of mercury in every direction. The perfect valves belong only to the branches and small trunks. Besides these imperfect valves, the vascular network contains here and there small pouches, which consist of dilated vessels, corresponding most frequently to their points of union. No apertures are discoverable in these vessels. Although, in their disposition, the lymphatic vessels have the greatest analogy with the blood-vessels of the skin, (for, as the latter, they form plexuses,) there is a point where the lymphatics pass the arteries and veins, and alone constitute the last layer of the plexus. Panizza found that, after having made a very fine injection of the lymphatics of the cutis, the epidermis might be removed without the escape of any mercury, and no traces of lymphatic vessels were visible in the membrane taken away.

MM. Breschet and Roussel de Vauzème employed various methods of injecting the cutaneous lymphatics: sometimes by introducing the tube into a lymphatic vessel of the leg, whence the mercury passed as far as the cutaneous system of the groin; at others, by passing the tube directly into the skin at the part where it was wished to examine these vessels, having previously injected the capillary system of the part, to avoid the danger of confounding the two orders of vessels. (Pl. II. Fig. 4, 5.)

There is an extensive plexus of lymphatics in the skin of the scrotum. Pl. II. Figs. 4, 6, represent these vessels in a portion of the scrotum, the prepuce, and glans of an infant. The removal of the epidermis, after an injection, never gave rise to the escape of mercury; and it was evident that the vessels distended by the mercury were distinct from those into which the colouring matter had passed. The lymphatic and sanguineous vessels appear to form distinct planes, situated in the substance of the mucous body, around the papillæ, the lamellated processes of the epidermis, and the sudoriferous canals. The inhalent vessels, in their empty state, appear to be the same organs as those into which, in other parts, the mercury has been injected. If, in their dry and empty state, their retiform arrangement has not been so easily recognized, it is owing to the circumstance that microscopic investigations could only be made on a small portion of the skin, and in which every vascular trunk was isolated by the rupture of its lateral anastomotic branches, some of the remains of which could sometimes be still recognized. The origin of the inhalents must be considered a point of anatomy still uncertain, and one which, if it has not been able to be decided on the surface of the intestines, presents still greater difficulties in the cutaneous system.

It is to be regretted that M. Gurlt has made no allusion to the vessels of the epidermis, which the French authors describe as inhalents. The existence of vessels in the epidermis has been previously maintained. Haller speaks of them as running along its inferior surface; but he suspected that, in the instance in which he had observed them, they might have been separated from the external surface of the dermis. The vascularity of a layer of the epidermis, described as the tunica quarta by some French anatomists, is very manifest in some injected preparations in the museum of St. Thomas's Hospital. There can be no doubt that these vessels belong to the sanguiferous system. Are they the same as those which the French authors have described as inhalents? This appears probable on several grounds; for they deny the existence of blood-vessels in this part, and describe the vascular system which we have lately noticed as subservient to the function of inhalation, for the three following reasons: (1,) because these vessels are prolonged to the most superficial layer of the epidermis; (2,) because their texture assimilates them to lymphatics; (3,) because absorption being a function of the skin, we find no other organ appropriated to this function. We may reply to the first argument in favour of their inhalent function by the question, Supposing them to be blood-vessels, why should they not be continued to the most superficial layer of the epidermis? The value of the second argument is easily to be estimated from what has already been quoted from the French authors, where it will be seen that they have endeavoured to explain why the lymphatics of the skin should *not* be like those of other parts of the body. The appearance of partial septa within these vessels is one which might be owing to an optical illusion beneath the microscope; and indeed it is difficult to imagine how so accurate an acquaintance with their extent, or even existence, could have been ascertained through the coats of vessels which are described as "white and silvery;" characters which are incompatible with much transparency. To the argument which is derived from the absorbent functions of the skin, and the absence of other organs appropriated to this function, it may be replied, that the existence of any inhalent function of the skin is by many physiologists denied; and those who maintain it have not always made due allowance for the fallacies to which their reasonings are exposed, from absorption in the lungs, the effects of friction, &c. It can only be admitted, from our present knowledge, that the skin is possessed of a very feeble absorbing power; and it is probable that this is exerted chiefly, if not entirely, in certain localities. But these inhalent vessels are said to exist in very great numbers, corresponding in fact to those of the perspiratory ducts. Admitting the existence of some degree of absorbent function in the skin, it cannot be said that organs do not exist independently of these supposed inhalent vessels, by which this function may be exercised. There is, as MM. Breschet and Roussel de Vauzème have shown, an extensive system of superficial cutaneous lymphatics. In order that matters from without may come in contact with these inhalent vessels, it is necessary to admit previous imbibition of the external surface of the epidermis; and it is only to suppose that this imbibition is a power possessed by the remainder of the same tissue to account for the mode in which any matters which are imbibed may come within the influence of the superficial cutaneous lymphatics.

There are other objections to admitting that these vessels are inhalents. Whilst reasoning on their functions, and as to whether they are the same as those into which the mercury was injected, it is said that the difficulty, in their empty state, of recognizing their reticular arrangement, was owing to the extremely small portion of skin which could be examined by the microscope, in which every vascular trunk was isolated by the rupture of its lateral anastomotic branches; and yet representations are given of entire vessels with their branches, the arrangement of which is totally different from that of the lymphatics, and much more like that which in other parts is given to arterial vessels. (Compare Pl. I. Figs. 8, 9, 10, with Pl. II. Fig. 4.) Thus, the arrangement must either have been seen, so as to admit of an inference being drawn from it, or the representation is imaginary. The former alternative would induce the belief that the vessels are not like lymphatics; the latter, that their existence is somewhat doubtful. The inference, too, that they are the same order of vessels as those which in other parts admitted the mercurial injections, is scarcely admissible, if, as is asserted, a coloured injection of the blood-vessels of the cutis might be pressed into them, whilst they never admitted the mercury. Of the nature also of the canals (Pl. I. Fig. 7, *a*,) with which these vessels are said to communicate beneath the papillæ, or of what system they might be supposed by injections to form a part, we are not very accurately informed. We think, therefore, that the functions of these vessels is a subject for further investigation.

CHAPTER V. *Mucific Apparatus, (Appareil blennogène,) or organ of the mucous matter.* It is necessary, in order to examine this well, to have a fresh skin, injected red with blood. In examining this from within outwards are said to be found—I. In the cutis, (1,) a mucific apparatus, composed of a secreting gland and an excretory duct of the secreted or mucous matter, which becomes epidermis by dessication; (2,) a colorific apparatus, composed of a secreting parenchyma and excretory canals of the secreted product, (scaly corpuscles,) which, uniting with the mucous matter, gives rise to the various tints of the skin, hair, horn, feathers, scales, &c. II. External to the cutis, and the result of the mixture of the two secretions: (1,) epidermis; (2,) hairs, feathers, horns, hoofs, &c.

1. Mucific apparatus. At the bottom of the dermis (Pl. II. Fig. 1, *j*,) may be seen small, reddish glands, which, when examined by a simple lens, appear crimpled, uneven, furrowed by blood-vessels, (Pl. I. Fig. 11.) They are enveloped in a loose cellular membrane, in an atmosphere of small adipose vesicles, transparent and accumulated like little pearls. From the summit of each gland passes a tube, which traverses the dermis and opens in the bottom of its furrows. This canal is enveloped in a diaphanous cellular membrane. Capillary vessels adhere to the tube and gland: at the base of the latter, a vessel of considerable size has often been seen to enter. The canals form a regular colonnade in the substance of the dermis. The glands are sometimes placed at very variable heights, and appear to communicate by intermediate ducts. The rows of the excretory ducts correspond to the length of the furrows; that is to say, they are perpendicular to the plane of the secreting parenchyma of the colorific organs.

2. Colorific apparatus. This is situated in the external part of the dermis, in the depths of its furrows, beneath and between the papillæ. (Pl. II. Fig. 1, *i*.) Its superior part is surmounted by a great number of short excretory ducts, which open at the bottom of the furrows, and which secrete a peculiar matter. (Pl. I. Fig. 13, *d*.) Its inferior surface is thickly set with capillary vessels, and in relation with the excretory tubes of the mucific glands. Its structure is areolar, spongy, resistant. This parenchyma and its excretory ducts grow red with great facility, because they are essentially vascular; they form a limit which, in the normal state, the arterial system never passes, and where it ceases to exist in bringing its last contribution. We except the nutritive vessels of the papillæ, which extend somewhat higher. When this tissue is torn, a number of little filaments are found, whence there escape scales or colourless corpuscles in great quantity. This reservoir of scales exists in no other part of the cutis. This tissue may be regarded as an organ formed of a peculiar substance, penetrated by blood-vessels, and giving off excretory ducts, which open at the same parts as those of the mucific glands, and pour into the mucus of these glands the granulations of pigment, or colouring matter properly called.

3. The products excreted constitute the epidermis, (Pl. I. Fig. 2.) Its inferior surface exhibits inequalities, which represent the form of the external surface of the dermis. This is the rete mucosum of Malpighi. Two partitions are here distinguished: one, more in relief, or dermic, filling the furrows of the dermis, and adhering to it by the prolongations issuing from the excretory ducts of the colorific and mucific organs. It is by these that the epidermis is produced and renewed. In separating the epidermis, a considerable resistance is always found when it is extracted from the furrows of the dermis, on account of the roots which it there seems to project, (Pl. I. Fig. 12, *d*,) although it is rare to perceive them, because it generally detaches itself as smoothly as if it were only placed in the depth of the furrow. On the lateral parts are seen small apertures for the passage of the inhalent vessels. The other partition, termed interpapillar, occupies the interval left by the bifid papillæ, and prolongs itself into the interstices around the sudoriferous and inhalent canals. On the borders of this, the torn fragments of the sudoriferous canals are always visible. To the right and left of this part are the sheaths, as it were, into which the papillæ penetrate obliquely. The epidermis which circumscribes these openings is fixed to the two partitions, which thus resemble a timber-work *(charpente,)* sustaining this curious structure. On the superior surface of the epidermis there exist prominent lines, separated by the furrows, (Pl. I. Fig. 1, E.) Under a lens, these lines present, alternately, small papillary eminences and fissures or slight depressions, which contain the orifices of the sudoriferous canals. The prominent lines have an imbricated arrangement, so that, in the movements of contraction, they advance over one another, as the scales of a serpent; whilst, during extension, they separate, and leave the bottoms of the furrows exposed. The human epidermis is of a dull white colour, elastic, hygrometric, and transparent. It is most difficult to examine satisfactorily. It rebounds under the scalpel like caoutchouc; when moist, it swells and allows nothing to be seen; and, when dry, it scales off and whitens, with the slightest contact or least degree of pressure.

The epidermis of the whale is more easily examined, and its structure throws much light on that of the human skin. The epidermis is secreted by a special apparatus, and appears to become organized like false membranes; hence the propriety of the term tissue. When examined by the naked eye from without inwards, the epidermis of the whale presents two layers; an external one, parallel to the plane of the cutis, and an internal one, composed of straight fibres, placed perpendicularly between the cutis and the external layer. Through this dark tissue the summits of the white nervous papillæ are seen enveloped by their sheaths. Its inferior surface contains openings for the papillary cones. To analyze this epidermis, it is necessary to take a very fine perpendicular fibre, and to place it in the focus of a lens upon a glass slightly moistened. The tissue is then found to consist of small, imbricated, scaly bodies, upon a very fine cellular woof. These scales readily separate, and tinge water of a black colour, under the appearance of granulations. Considered singly, each scale has the form of a fig or of a spatula with blunt edges. Its two surfaces are black in the upper third of their extent; its free border somewhat rounded, its pedicle contracted and whitish. In order to study it, it is necessary to take a small quantity of the dark matter at the base of the fibre near the dermis, and to stir it in a few drops of water upon a glass. A fibre of epidermis, reduced to its most simple state by dissection, and examined by a microscope, is found to consist of a series of scales or flattened cones, inserted one into the other. Each scaly portion is applied upon that which follows, and is covered somewhat by that which precedes it, in the same manner as a pine-apple. This fibre is elastic and somewhat resistant, but its component parts separate, and may thus be examined. The points of the origin of the epidermis are very well seen in the skin of the whale, on account of the contrast between its dark colour and the whiteness of the dermis. It fills the entire space which is unoccupied by the papillæ. The dark matter is secreted a little previous to its appearance external to the dermis. It is found enclosed in a capsule or dermic membrane, at the bottom of which are little whitish or filamentous tubercles, to which it is closely united: these are the excretory ducts of the colorific apparatus. This development is from within outwards. The matter, which is at first formed almost in a mucous state, pushes forwards the superior layers, which gradually solidify; and this takes place by a successive expulsion of scales and mucus, the external layers of which are always the oldest, the most compact, and the least distinct. From our knowledge of this elementary form, it is easy to derive all the other forms which the epidermic tissue presents. If several of these fibres proceed from the cutis in a straight line, and compressed against each other as in a hurdle, we have a membrane; disposed circularly, a cylinder, a sheath of papillæ, a case to protect them. If the fibres arise, "denso agmine," a thick compact tissue is the consequence, which will fill the intervals of the papillæ. If, arrived at a certain height, these fibres curve at an angle more or less obtuse, the body will be stratified parallel to the dermis; for dissection shows that the layers are produced by the inflexion of the perpendicular fibres. The sheath which this matter furnishes to the papillæ is formed of a tissue less white than the nervous trunks. It is greyish, and the scales are less numerous and less coloured than in the epidermis proper; there is preponderance of the cellular or mucous woof. This sheath moulds itself perfectly around the papillæ. In the horizontal layer, the scales are more compressed, and very difficult, if not impossible, to detach: hence this portion, although very dark, does not tinge water, because it is not there dissolved into squamules. The increasing and always more intimate adherence of the external layers one with another explains the formation of the numerous epidermic layers, which separate in consequence of maceration, and in which the imbricated form is sufficiently manifest.

Having examined the structure of the epidermis of the whale, the French authors proceed to that of man. A friable portion of the most external part of the epidermis, or of the glutinous mucus of its internal surface, should be placed in water and examined by a lens. In separating these fragments with the point of a scalpel, in the midst of the remains of the inhalent vessels and sudoriferous ducts, an infinity of corpuscles are seen, which, either from the violence employed or from their union with one another, are without determinate shape. The general form of these scales is an irregular trapezium; they are of a certain thickness, striated, white and transparent, and placed upon a very thin areolar membrane. These scales are easily recognised as the product of the colorific apparatus, and, in the pellucid membrane which supports them, the secretion of the mucific apparatus is no longer amorphous. In order to see the origin of the epidermis in the furrows of the dermis, it is necessary to prepare a fine slice of the dermis, injected red, and it will be seen that where the blood ceases at the excretory ducts, the deposition of epidermis commences. This separation is very manifest in the whale. The epidermis, at first secreted mucous and fluid, moulds itself, layer after layer, around the papillæ, envelopes and protects the sudoriferous canals and inhalent vessels, after having acquired a density greater as it approaches the surface. If a portion of the skin of the heel has been sufficiently macerated, and is then cut across the furrows, layers of epidermis are seen to issue from the centre of the furrows, and to develop themselves right and left on the papillæ which they envelope. (Pl. II. Fig. 1, *l.*) The two tissues are developed in the same manner as in the whale, the difference consisting only in a variety of form. The epidermis in the negro is everywhere black, excepting in the palms of the hands and soles of the feet. Its structure is the same as in the white; in the black part of the skin the scales are in spatulæ, coloured on the free border as in the whale. On the heel, their form is an irregular polygon, and they are colourless. Examined by a lens, the skin of the rest of the body does not appear entirely black: it is seen that the colouring matter produced around the papillæ sketches them by forming areolæ, the centre of which appears to be white, because the papillary tissue appears through the transparent epidermis. The areolar woof which supports the scales is always white.

This concludes the account given by MM. Breschet and Roussel de Vauzème of the structure of the skin. The remainder of their work is devoted to the consideration of different opinions respecting the nature of the epidermis and colouring matter, together with a theory of the cause of the various colours presented by the skin and its accessory parts, which we shall briefly notice, after having considered the objections offered by M. Gurlt to the existence of specific organs for the secretion of the epidermis; objections with which, for additional reasons, we are disposed to coincide. In speaking of the epidermis, M. Gurlt notices particularly its intimate union with the whole dermis. He mentions also the granular appearance of its internal surface, from which Wendt inferred that the outer layers were not produced by simple dessication of those within. This difference is not considered as essential, but as temporary, the outer layers being formed by the breaking down or union of these granules. M. Gurlt has been unable to discover the apparatus to which the function of secreting the mucus is ascribed; but, as he is aware that he may be in error, he adduces the following reasons for believing the observations of the French authors to be erroneous. (1,) The supposed glands are, in the plate in which they are represented, very similar to the sudorific glands, in regard to position, figure, and size; and the excretory ducts want merely the spiral form, which form, however, is often absent even in the sudoriferous canals in the cutis. (2,) If the separated layer of the skin be of considerable thickness, we find in it twice as many sudorific glands and ducts as in a thin lamina, and as are represented in the figures of the French authors, and the more deeply seated canals appear to stop at the space between the cuticle and the corium; but, if we compress the lamina a little, they appear also distinctly in the cuticle. (3,) There does not appear to be sufficient space for the discharge and spreading out of a fluid (the mucus of Malpighi,) secreted by glands only; seeing that the dermis and epidermis are so closely connected. The difficulty is removed by supposing that the mucus is secreted by the whole dermis.

In addition to the arguments brought forward by M. Gurlt, we may notice the peculiar views of MM. Breschet and Roussel de Vauzème respecting the limits of the arterial system of the skin; as, if they are correct, the secretion of the epidermis, whether through the medium of a glandular apparatus or not, must take place entirely from the furrows of the dermis. When speaking of the organs of the colouring matter, which are said to be situated in these furrows, they say that, with the exception of the nutrient arteries of the papillæ, the parenchyma, and the excretory ducts of the colorific apparatus, form a limit, beyond which, in the regular state, the arterial system never extends, and where it ceases to exist in bringing its last contribution. If this were the case, injections would only redden the furrows, and fill the vessel or vessels of the papillæ which they describe as terminating by an arch in their substance. We have already noticed the opinions of many anatomists with respect to the extreme vascularity of the papillæ; a condition which induced Gaultier, who appears not to have perfectly understood their structure, to term them "*bourgeons sanguins.*" Beclard says, that "the vessels divide and ramify in the dermis in proportion as they penetrate into its substance, and their last divisions, which are prodigiously multiplied, are distributed in the external surface of this membrane and in the eminences which cover it, in consequence of which these parts are much more vascular than the deeper surface." (*Elem. of General Anat., translated by Knox.*)

The French authors, when speaking of the investing membranes of the papillæ, mention a process of the external surface of the dermis which is continued over them, but they say nothing respecting its vascularity: Eichhorn, who, in the *Archiv. für Anatomie und Physiologie, von Meckel,* 1827, has entered with great minuteness into the structure of the dermis, and who, from certain peculiarities in its structure, has described it as consisting of three different layers, which are however inseparable, calls the external layer, from its peculiar vascularity "*tunica vasculosa externa corii.*" The examination of the exterior of a successfully injected portion of dermis shows an almost entirely coloured surface, with no such limitations as those mentioned: and, in opposition to the limits which the French authors have ascribed to the sanguineous system, we may instance an injected portion of the scrotum of an infant, from which one of their drawings is taken, in which, among the superficial cutaneous lymphatics, are ramifying arterial vessels, (Pl. II., Fig. 4.) And, indeed, there is an equal discrepancy in their own descriptions of the limits of the arterial system of the skin. In that part of their work in which the superficial cutaneous lymphatics are described, they say that there are differences in the arrangement of the sanguineous vascular plexus and the plexus of lymphatic vessels, which prevent their being confounded; that these vessels appear to form distinct planes, situated in the substance of the mucous body (that is, the internal portion of the epidermis,) around the papillæ, the lamellated processes of the epidermis, and the sudoriferous canals. This is quite at variance with their description of the arterial system, when speaking of the colorific apparatus, but probably nearer the truth. On these accounts we are disposed to coincide with the opinion generally maintained, that the formation of the epidermis is dependent on the whole external surface of the dermis.

M. Gurlt has been likewise unable to discover the colorific apparatus. He says that, in the parts mentioned as containing these organs, he can recognize a stratum of a darker colour than the rest of the dermis; but he cannot discover, on tearing asunder this texture, the small scales mentioned by the French authors, nor the general peculiarities ascribed by them to this tissue. He therefore (although with diffidence,) expresses his doubts, which he considers to be further justified on the following grounds: (1,) The colorific organs are said to exist even in the skin of white men; but, in them, the epidermis is colourless. (2,) In all organs where there exists a brown-black or other pigment, as in the eye, no similar apparatus is perceptible. (3,) In morbid discolorations, as in melanosis, there is, likewise, nothing of the kind.

The first of these can scarcely be admitted as an objection, as the French authors state that, in the white, the scales are white and transparent; and, unless the identity of the colouring matter of melanosis and the skin be shown, there is little force in the third objection. But there are two facts which appear to us almost conclusive against the existence of this colorific apparatus. (1,) MM. Breschet and Roussel de Vauzème mention, as corroborative of their opinions respecting the mode of production, seat, and organic disposition of the colouring matter, that MM. G. Cuvier and Ch. Valencienne attribute to the cutis the property of secreting beneath the scales a substance of silvery brightness, which is the cause of the brilliancy of some fish; that it consists of small polished laminæ like burnished silver, and that, in many fish, much of this matter is secreted *in the substance of the peritoneum,* and in the coverings which it furnishes to various viscera, and particularly to the swimming bladder. (2,) The colouration of the hair is considered as depending on the same secreting parenchyma as that of the skin; and, in the latter part of their work, the French authors say that the hair originates in a layer much deeper than that which furnishes the pigment, and that that portion of it which is nearest to the bulb is never coloured. They say nothing of the colour of the bulb itself. Pl. II. Fig. 1, *i,* shows the limits of the supposed colouring apparatus; Pl. II. Fig. 2, *d,* represents the depth of the hair follicle and the situation of the bulb. Wendt thus describes the formation of the hair. In the fœtus we see the blackish germ of the hair confined in the sacciform follicle, whose cavity it does not entirely fill. It forms, in the bottom of this, a little club, the thicker part of which, the bulb, is directed towards the bottom of each sack; the point externally. But before the little club shows itself, a vessel is seen, which proceeds to the bottom of each sac, deposits a point of dark pigment, which gradually, by the accession of new pigment, becomes the hair bulb. Thus, it is evident that the bulb is coloured; that its pigment is formed far below the limits of the supposed secreting organs of colour, the bulb of the hair being seated in the subcutaneous adipose tissue; and that this pigment is formed without the intervention of any such secreting apparatus as that described by the French authors.

We are therefore disposed to hesitate before we admit the existence of the colorific apparatus of MM. Breschet and Roussel de Vauzème, and to ascribe the secretion of the colouring matter of the skin to the vessels of the external surface of the whole dermis.

It will have been seen that the rete mucosum is only the internal portion of the epidermis. The epidermis is secreted fluid, and this fluid is identical in its composition with its hardest portions. It becomes solid gradually, and in proportion as it is separated, layer after layer, from its origin. The scarious wings of butterflies are but mucous points in the chrysalis; and it is the passage from the fluid to the solid state which has been considered in man as a peculiar substance, or the rete mucosum; but this rete does not exist independently; it is but the expression of the transition state, and the form which the substance receives from the inequalities of the dermis. MM. Breschet and Roussel de Vauzème compare the rete mucosum in its relations with the epidermis to recently melted wax, one half of which is liquified by heat, the other condensed by the external cold.

The theory which the French authors have offered of the cause of the colours of the skin is founded on the existence of the small corpuscles, the formation of which they have attributed to a special apparatus. Gurlt states his inability to detect these bodies within the secreting parenchyma which has been described, but he does not say whether he was able to see them external to the dermis. He speaks of the granular texture of the inner part of the dermis. May not this be the same as that which is spoken of by the French authors as consisting of small scaly corpuscles? There are other opinions in favour of the existence of such bodies. We have already noticed the small polished laminæ described by G. Cuvier and Valencienne beneath the scales of some fish. MM. Breschet and Roussel de Vauzème remark, that, if the skin is black or white, the free border of the scales is coloured black or white. The pedicle of the scale and the cellular woof in which it is inserted are always of a white colour, as well as every part which enters accidentally into the composition of the epidermis: the scales are therefore the only seat of the colouring matter. They compared this structure with the wings of the lepidoptera, which are an epidermic secretion. The scales of butterflies, coloured and pediculated, are implanted in a species of central moulding, from which it is considered a fair inference that the fine plexus to which the scales of the human skin adhere is also a basis containing the canals proper to the scales, which is evident in the whale. The French authors found that the rich and varied colourings of flowers were the result of a chequer-work of small utricles of different forms and colours according to the species; and, as far as a comparison may be drawn between the animal and vegetable kingdoms, they suppose that the seat and mode of coloration are analogous in both. They imagine that the form of the scale, or utricle, may have some influence in the production of the colour; that the connexion of the pedicle of the scale with its secreting organ is a medium through which it is nourished by a fluid circulation. The differences of colour of particular races, they suppose to be dependent on the different forms of their scales, and that there is no necessity to attribute it to the influence of the sun, which, although it may more or less tan the skin, has not the power to change the primitive type of animals. But, as this arrangement of little scales could only constitute difference of form, it is always necessary to admit some peculiar colouring matter, the supposed organ of which has been described. The dermis is white, or is only coloured beneath by the vascular network which has been mentioned. All the organs which arise from it are colourless; but, at the limit of the secreting tissue, the production of colouring matter is seen, and coloured globules have been observed in what are deemed the excretory canals of this tissue. Thus, a peculiar modification of the mucous substance takes place in this glandular parenchyma, consisting in the addition of a colouring matter of various characters, showing that the mucous and colouring principles are distinct, although never isolated from one another when constituting the epidermis.

(It appears sufficiently probable, that, inasmuch as the colours of the skin are said to depend on the presence of certain corpuscles, there is reason in this theory. Much, however, respecting the nature of these scales is purely hypothetical, and we have already stated our doubts as to the existence of the supposed colorific apparatus.)

From what has been said of the structure of the epidermis, it is evident that it is not an inorganic substance; it is, on the contrary, a tissue of a somewhat complex organization, connected with the important functions of exhalation and absorption by the faculty of allowing the passage of liquids: but its vitality would appear to be on a par with that of vegetables. The absence of nerves proper to it renders it insensible; it is coloured, exhales and absorbs in the manner of vegetables.

In another part of their work, the French authors say that the epidermis appears to become organized like false membranes: but false membranes are more highly organized than vegetable matter. The doubts which exist as to the nature of the vessels in the epidermis termed inhalents leave still room for question as to the degree of vitality and organization of this tissue.

The sebaceous glands and hair follicles are minutely described by

Gurlt. He has been more successful than both Gaultier and Weber in examining these organs. The former stated that the neck of many hairs was surrounded by glands. The latter speaks of them as consisting of acini, and mentions his having seen in two instances a hair, the root of which projected beyond the divisions of the gland, so that it was situated in the cellular tissue beneath the skin. (*Archiv. von Meckel,* 1827.) M. Gurlt, who has been more successful in his mode of examination, thus describes them.

They are processes of the epidermis penetrating the dermis. The glands and follicles are usually united; at least, where there are hairs there are always glands; but there are sometimes glands where there are no hairs, and, in the palms and soles of carnivorous animals, both are wanting. The sebaceous glands (Pl. II. Fig. 2, *g*,) lie very superficially in the dermis; and in this they are distinguished from both the hair follicles and sudorific glands, both of which always penetrate the lowermost layers of the dermis, and often project beyond them. The sebaceous glands vary in form and size in the same animal, and there are many varieties of them in different animals. They are generally oval, consisting of small acini, which are transparent when they do not contain fat. They generally resemble clusters of conglomerate glands, and are consequently improperly termed follicles. The excretory ducts of the glandular acini either unite so as to constitute a single duct, (Pl. II. Fig. 2, *h*,) opening in the capillary pores, (as is the case with the smaller glands;) or several ducts, frequently from four to six, enter together into one pore. Where, however, the sebaceous glands exist, where there are no hairs, as in the prepuce and glans, then the common excretory duct opens immediately on the cuticle. Two glands are commonly connected with one capillary pore, and one is always found. They are larger where the hair is thicker than where it is thin. They are larger in the hair of the head in man than in the hairy coat of animals. The sebaceous glands are discoverable with the naked eye on the cut surface of recent skin, near the junction with the epidermis, and resemble white granules. In order to examine them with the microscope, a thin lamina of the dermis must be cut in the direction of the hairs; since, where they have a sloping direction, a perpendicular section of the skin must cut through both the glands and the hairs. If the separated lamina is not thin enough, a moderate degree of pressure makes the glands more perceptible, by pressing out the fat: in doing this a little water must be added, to prevent the lamina from sticking to the glass or being torn.

The hair follicles (Pl. II. Fig. 2, *f*,) are by some termed hair-bulbs, but this term is only applicable to the enlarged portion of the hair itself, (Pl. II. Fig. 2, *d*;) the whole hair being divisible into this, the shaft and the point. These follicles are produced by the dipping down of the epidermis, as can be proved in the slightly macerated skin of the fœtus, by a careful separation of the epidermis from the cutis. In this case, we see the hair follicle remain on the under surface of the epidermis, with its rudimental hair enclosed; but, if the hair is somewhat more grown, then we find the follicle commonly torn, as always happens in the case of the perspiratory ducts. The follicle is evident at its lowest shut extremity where it enters the dermis; it becomes narrower as it penetrates the epidermis, and so closely encloses the hair when it has passed through this, that it seems to be united with and lost in it. But this is not the case; since, if we press the sebaceous matter from the glands into a capillary follicle, we find it escape along the hair on the free surface of the epidermis. By using a great magnifying power, we can discover in the bulbs of the hair thin fibrils, which are not unlike the capillary rootlets of plants, and which probably absorb nourishment from the parts into which they are implanted. The office of the hair follicle is evidently the formation of the hair, just as the capsule forms the tooth: it receives the fat secreted by the sebaceous glands, and conveys this along the hair to the cuticle, at the same time oiling both.

The analogy which is here drawn between the formation of a tooth from its capsule, and the hair from its follicle, must be taken with some limitation; since, in the former case, the secretion takes place from the whole surface of the capsule; whereas, in the latter, as M. Wendt remarks, the growth of the hair is from the bottom of the follicle; and, in the fœtus, the rudimental hair lies loose in its sheath, which it does not entirely fill, the course of the nutritive vessel being towards the bottom of the sack.

On the subject of the Pathology of the Skin the French authors have said but little, and that little is purely speculative. They, however, purpose to make it an object of enquiry; and, if founded on a correct anatomy, we may trust that the many difficulties, both of diagnosis and treatment, which, notwithstanding all that has been hitherto effected, still encumber this subject, will be in a great measure removed.

The amount of information which we can satisfactorily derive from the works of which we have now completed the notice, although valuable, is considerably less extensive than their pretensions. It would appear that the descriptions which are given of the organs of perspiration, of the sebaceous glands and hair follicles, and of the identity of composition of all parts of the epidermis, may be depended on. But there is reason to doubt whether a somewhat too limited view has not been taken of the structure and functions of the cutaneous papillæ; whether any organs exist to which are exclusively appropriated the secretion of epidermis and its colouring matter; and whether a correct function has been ascribed to certain vessels within the epidermis. With regard also to the degree of organization of this tissue, there is still ground for question. The impression produced by a first perusal of the essay of MM. Breschet and Roussel de Vauzème is, that they are indebted to their imagination for some of their facts; and a more careful examination has but confirmed this suspicion. We may express a hope that, endowed as they are with the faculty of minute and persevering observation, a similar remark may not be applicable to their promised investigations of mucous membranes. There has been less of the search after novelties in the works of the German authors; their investigations having been chiefly directed to the structure of parts, the existence of which had been previously ascertained.

---

### Art. VII.

*Explanation of the Plates to illustrate the Article on the Structure of the Skin, p. 429. Plate I.*

Fig. 1. (In this drawing, the component parts of the skin are represented simply with respect to their relative situations and forms, without any regard to their number.) Inferior surface of the foot. A. External surface of the epidermis of the heel. *b*, prominent papillary lines separated by transverse fissures, *c*; in the middle of which is seen the opening of a sudatory pore. *d*. Furrows parallel to the prominent lines. E. Internal surface of the epidermis, moulded upon the dermis and lifted up. *f*. Series of apertures which receive the papillæ. *g*. Little interpapillary partition, or prominence of epidermis, interposed between two bifid papillæ, and pierced by apertures for the passage of the sudoriferous canals, some of which are seen (*h*) in the form of threads penetrating into the dermis. *i*. Large partition of the epidermis, more in relief than the former, and received into the furrows of the dermis. *j*. External surface of the dermis. *k*. Prominent lines abounding in papillæ, generally two and two, and between them (*t*) the apertures by which the sudoriferous canals pass outwards and the inhalent vessels enter. *m*. Furrows of the dermis, where the excretory canals of the epidermic matter terminate. N. Internal face of dermis, perforated for the passage of blood-vessels, nerves, &c. O. Adipose tissue beneath the dermis.

Fig. 2. Portion of the internal surface of the Epidermis, which is in contact with the dermis; the same as that marked E in the preceding figure, but much magnified and dried. An upper layer of matter has been removed, that the perforations may be better seen. *a*. Prominent septa received into the furrows of the dermis, perforated laterally by small apertures for the passage of the lymphatic vessels. *b*. Interpapillary septa, pierced by the sudoriferous canals. *c*. Apertures which serve as sheaths to the papillæ.

Fig. 3. *a*. A group of papillæ, magnified. *b*. Dermis.

Fig. 4. Sudoriferous organ. *a*. Dermis. *b*. Glandular secreting organ. *c*. Spiral excretory canal, passing between the papillæ, traversing the epidermis, and opening at the pores of the skin.

Fig. 5. *a*. Nervous papillæ. *b*. The same cut near the dermis, and turned so as to exhibit the opening which corresponds to each papilla, by which the nervous pulp and the blood-vessels enter.

Fig. 6 represents the apparatus which constitutes the tactile organ in man. *a*. Nerve entering into the dermis, where it becomes capillary. *b*. Its entrance into the papillæ. *c*. Neurilema furnished by the dermis. *d*. Proper envelope of the nerve. *e*. Layer of epidermis, more or less thick, the organ of protection.

Fig. 7. *a*. Canal of the inhalent vessels. *b*. Papillæ. *c*. Epidermis. The branches which come from the epidermis terminate in the common trunk.

Fig. 8. Inhalent vessels in the epidermis of man.

Fig. 9. A fragment of these vessels, farther magnified.

Fig. 10. Inhalent vessels, such as they have been seen upon the epidermis of the trunk of an elephant.

Fig. 11. *a*. Secreting organ of the mucous matter. *b*. Its excretory canal; *c*, blood-vessel. *d*. Small, whitish granules which surround it.

Fig. 12. *a*. Dermis. *b*. Papillæ. *c*. Epidermis lifted up, at *d*, to show its origin in the furrows of the dermis between the papillæ. The torn prolongations correspond to the excretory ducts of the colorific apparatus.

Fig. 13. *a*. Colorific organ, torn in two parts (*b* and *c*,) to show the escape of the scales which are there formed, and the filiform vessels of which the organ is composed. *d*. Small excretory canals, which are torn where the epidermis is elevated. *e*. Secreting organ of the mucus, which is poured out above the colorific organ. *f*. Fluid state of the epidermis, i. e. pigment or scales floating in the midst of the mucus. *g*. Layers of epidermis, which are stratified to the right and left, and which become more condensed in proportion as they become more external.

### *Explanation of Plate II.*

Fig. 1. A synthetic figure or schema of the Human Skin. *a.* Dermis. *l, m.* Epidermis. c. Vessels and nerves which enter into or which escape from the dermis. *e.* Nervous papillæ. *f.* Sudoriferous organ. *g.* Its spiral excretory duct, which traverses the dermis, passes behind the papillæ, and terminates at the pores of the epidermis. *h.* Inhalent vessels, originating in the most external layer of the epidermis, ramifying and anastomosing before they penetrate the dermis by the openings through which the spiral ducts pass. *i.* Colorific organ. The portion which is cut is alone seen, because it extends along the furrows. Its excretory canals open in the furrows, between two ranges of papillæ. *j.* Organ which secretes the mucus. *k.* Its excretory canal, opening in the furrows of the dermis between the papillæ. There the mucus, mixed with scales, at first fluid, solidifies in successive layers, right and left, as is seen on that incision of the skin which is made across the furrows, *l*; but, in the longitudinal section, *m*, these layers present a series of straight lines placed above one another. It is thus also that the epidermis is decomposed by maceration. The superior surface of the epidermis presents furrows, *n*, which correspond to those of the dermis, and the prominent papillary lines, *o*; separated by transverse fissures, *p*, at the bottom of which are found the pores of the sudoriferous canals.

Fig. 2. A lamina of the skin of the human head. *a.* The epidermis, here very thin. *b.* The dermis. *c.* The adipose tissue beneath. *d.* The hair bulbs. *e.* The shaft of the hair. *f.* The hair follicle. *g.* The sebaceous glands; *h*, their duct. *i, k.* Sudorific organ, (Gurlt.)

Fig. 3. A perpendicular lamina of the skin of the human palm. *a.* Outermost layer of the epidermis. *b.* Middle layers of the same. *c.* Mucous tissue of Malpighi. *d.* Papillæ. *e.* Dermis. *f.* Adipose tissue. *g.* Sudorific glands. *h.* Ducts of the sudorific glands.

Fig. 4. Skin of the scrotum of an infant. *a, a.* Lymphatic vessels; *b, b*, arteries. (Gurlt.)

Fig. 5. Portion of the skin of the adult scrotum. *b, b, b.* Plexus of lymphatics injected with mercury, and exposed by the removal of the first epidermic lamina. *a, a, a.* The same plexus, with the lymphatic vessels which pass to it, to form it. Here the epidermic lamina has not been removed; here and there a few hairs are seen which pass from the skin. A straight line (*c, c*,) indicates the cut of the epidermis.

Fig. 6. The penis of an infant: the prepuce is divided. *a, a.* Lymphatic vessels of the skin of the penis, communicating with the plexus of the prepuce. *b, b.* Plexus of lymphatic vessels, injected with mercury, of the skin of the prepuce, seen on its internal surface. c. The same vessels of the glans.

Fig. 7. One of the aspects presented by the human dermis beneath a magnifying glass, when cut parallel to its furrows. *a.* Blood-vessels covered with capillary filaments penetrating the dermis. *b.* Nerves becoming capillary. *c.* Mucous glands, placed at unequal heights, and anastomosing with one another. Their excretory canals penetrate as far as the epidermis. *d.* Spiral sudoriferous canals. *e.* Fragments of vessels. *f.* An infinitude of vessels or of capillary nerves. *g.* Colorific apparatus, surmounted by its excretory canals. *h.* Papillæ.

## Figures and Tables

**Fig. 1 F. 2. Fig. 3 Fig. 4. Fig. 5. Fig. 6. Fig. 7. F. 8. F. 9. F. 10 Fig. 11. F. 12. Fig. 13. f1:**
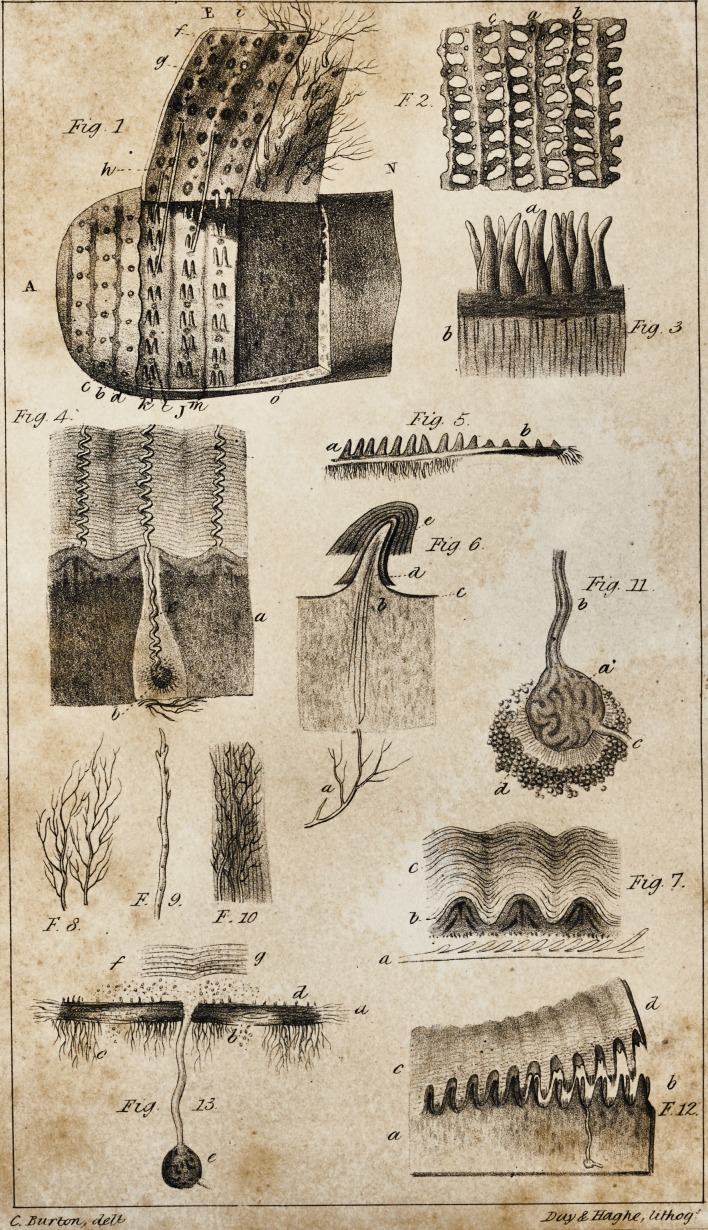


**Fig. 1. Fig. 2. Fig. 3. Fig. 4. Fig. 5 Fig. 6. Fig. 7. f2:**